# The reference dependence roots of inaction inertia: A query theory account

**DOI:** 10.1371/journal.pone.0282876

**Published:** 2023-03-31

**Authors:** Ruth Pogacar, Neil Brigden, Emily Plant, Frank R. Kardes, James Kellaris

**Affiliations:** 1 Department of Marketing, University of Calgary, Calgary, Alberta, Canada; 2 Department of Entrepreneurship, Marketing and Social Innovation, Mount Royal University, Calgary, Alberta, Canada; 3 Department of Management and Marketing, University of Montana, Missoula, Montana, United States of America; 4 Department of Marketing, University of Cincinnati, Cincinnati, Ohio, United States of America; Universitat Jaume I Departament d’Economia, SPAIN

## Abstract

Inaction inertia is the tendency to forego an opportunity after missing a significantly better opportunity. We show that inaction inertia is rooted in reference dependence. This is consistent with prior work finding that smaller discounts are devalued and inertia is motivated by avoidance of loss. We further illuminate the process by showing that consumers treat the missed discount (rather than the regular price) as a reference point relative to which a smaller discount feels like a loss. Missing a significantly better deal causes people to focus first and foremost on thoughts critical of the current deal. Notably, consumers who miss a smaller discount also construe the second deal as a loss, even if they take it. This research integrates inaction inertia and reference dependence theory using query theory analysis to contextualize inaction inertia with biases such as loss aversion, anchoring, and the default effect.

## 1 Introduction

The literature on consumer biases is rapidly expanding [1]. However, despite identifying ever more biases, research has thus far developed little unifying theory to understand common mechanisms [2]. Moreover, many decision traps, such as inaction inertia, have not been integrated with judgment and decision-making theory. It is imperative for the advancement of science that the subfields of psychology develop cohesive theory to understand the common roots of human biases. The present research seeks to bridge the literatures of consumer behavior and judgment and decision-making by showing that reference dependence drives the inaction inertia bias.

## 2 Inaction inertia

Inaction inertia refers to the tendency for decision makers to forego an opportunity because it is similar to, but substantially inferior to, a previously missed opportunity [3, 4]. In the classic illustration, people who are asked to imagine that they can buy a special pass to their favorite amusement park for $90 instead of the usual $100 price generally want to take advantage of the 10% savings. So do people who imagine that they missed a small opportunity to buy the pass for $80 but can still take advantage of the $90 offer. However, when told to imagine that they missed an opportunity to buy the special pass for $40, people are significantly less likely to take advantage of the current $90 offer. This has perplexed economically minded researchers because missing the initial offer does not diminish the absolute value of the current offer. Inaction inertia can lead to non-optimal decisions from both a consumer and firm perspective in that consumers may avoid desirable current purchase opportunities because of previously missed opportunities. Furthermore, prior research has shown that inaction inertia can even drive customers away from the firm’s products and into the arms of competitors [5].

While the inaction inertia phenomenon has been replicated in multiple domains [3, 4, 6–18] there is not a consensus on the psychological mechanism underlying the effect. Prior research suggests that inaction may be attributed to the motivation to reduce feelings of regret from missing a superior offer [3, 6], or that it occurs because people devalue subsequent offers [6, 11, 17]. However, the question remains: *why* do people engage in biased reasoning after missing an initial opportunity and how might these cognitions integrate with judgement and decision-making theory more generally?

### 3 Reference dependence and query theory

We propose that inaction inertia is rooted in reference dependence, the tendency for people to evaluate prospects as gains and losses relative to a reference point [19]. Reference dependence is the mechanism underlying many consumer decision traps [20], including anchoring [21], status quo bias [22], and the default effect [23]. For instance, anchoring occurs when people focus on initial information, such as a price, as the reference point, and subsequent information is judged as a gain or a loss relative to the reference point. This creates bias because prices that are lower than the anchor seem reasonable, even if they are higher than market value, whereas prices above the anchor seem less reasonable, even if they are below market value [21]. If inaction inertia is also driven by reference dependence, it would be similar to anchoring in that the subsequent offer is evaluated relative to the reference point of the initial offer. Notably, the anchoring literature emphasizes the lack of adequate adjustment when evaluating information relative to an anchor.

Not all reference points are equally influential. Prior work on inaction inertia has attempted to rule out accounts based solely on the contrast between the current and previously missed offers. For example, Tykocinski et al. [4] showed that if the missed deal was “back home in Canada” rather than where the participant was currently located, no inaction inertia was observed. Tykocinski and colleagues therefore concluded that inaction inertia cannot be attributed to contrast effects (i.e., reference dependence) alone. Thus, not just any other offer will serve as a reference point and produce inaction inertia—the missed opportunity must have been a *very similar* opportunity in order to serve as a reference point in evaluating the current opportunity. Only then is inaction inertia likely to occur.

Similarly, Arkes et al. [6] tested an anchoring/devaluation account for inaction inertia. They found inaction inertia when a previous offer was available in nearby St. Louis, or Columbus, but no effect when the prior offer was in South-East Asia. These results are again consistent with the theory that the impact of a prior opportunity on evaluations of the current opportunity depend on the similarity of the two opportunities and the possibility that the prior opportunity could have easily been taken. It was further proposed that devaluation arising from a negative contrast between the current and foregone offer may drive the inaction inertia effect.

## 4 Hypotheses

According to our theorizing, devaluation of the current offer in inaction inertia scenarios occurs because reference dependence leads people to focus on the advantages of the missed deal, and therefore construe the current deal as a loss rather than a gain. This shift in focus is consistent with the query theory account of the way people focus on the advantages of the status quo and disadvantages of change from the status quo [24]. Thus, when people are told they have missed a much larger discount (e.g., 60% off), and are subsequently offered a significantly smaller discount (e.g., 10% off), they construe the foregone deal as a reference point to which the current deal seems like a loss. Conversely, people who have *not* foregone a significantly superior opportunity construe the full price option as the reference point, to which the current deal seems like a gain. We predict that:

H1: Participants who have missed a significantly better deal will focus on a lower reference point than participants in the small missed deal or control conditions. Therefore,

H2: Participants who have missed a significantly better deal will construe the current deal as a loss, whereas those in the small missed deal and control conditions will construe the current deal as a gain.

In their seminal account, Tykocinski and colleagues [4] propose that thinking about the significantly better, missed deal produces an unpleasant experience of regret; therefore, people forego a subsequent, smaller deal in order to put an end to the aversive regret. According to this account, inaction inertia is a strategy to avoid thinking about the loss of the previous offer. We propose that reference dependence makes the *current* offer, rather than the foregone offer, feel like a loss.

More recent research posits that people who miss a significantly better deal *place a lower value* on the subsequent opportunity than those who only missed a slightly better deal, and this devaluation causes inaction inertia [6, 17]. While consistent with our theorizing of how the current deal is construed as a loss, this account does not integrate reference dependence into the process. Moreover, Zeelenberg et al. [17] measured the devaluation of the second offer in terms of absolute value–i.e., how much consumers would pay for the second offer relative to the first–rather than whether the second offer was construed as a loss, as we propose. To examine whether the current deal is construed as a loss we test a query theory process account.

Query theory provides a process account for people’s biased recall of thoughts regarding the pros and cons of endowed (reference) options versus the alternatives [24]. According to query theory, people focus first and foremost on the pros of the focal option and cons of the alternative; only secondarily, if at all, do people think about the pros of the alternative and cons of the focal option. Thus, we propose that when a consumer is told that he or she has missed a significantly better offer, the superior missed deal functions as a reference point. When the person is offered a similar but less desirable offer they call to mind first and foremost thoughts favoring the reference–foregone–deal and critical of the current deal. Only secondarily, if at all, will people generate thoughts favorable of the non-reference–present–deal and critical of the foregone deal. Therefore, a current discount on a desirable product may be evaluated unfavorably relative to a significantly better discount that was previously available but missed. The current deal may be construed as a loss rather than a gain, making consumers less likely to take advantage of the currently available discount. This theorizing is consistent with recent findings that inaction inertia is partly due to a focus on past opportunities [25]. Hence:

H3a: Participants who have missed a significantly better deal will be more likely than those who have missed a smaller deal or those in the control condition to list thoughts favorable of the foregone deal and critical of the current deal.

H3b: Participants who have missed a significantly better deal will be more likely than those who have missed a smaller deal or those in the control condition to list thoughts favorable of the foregone deal and critical of the current deal *before* listing thoughts favoring the current deal or critical of the foregone deal.

Finally:

H4: Reference point focus will mediate the effect of missed deal on gain/loss frame.

H5: Thought focus and thought order will mediate the effect of missed deal on likelihood of purchase.

Identifying a reference dependence mechanism would situate inaction inertia in the context of consumer biases such as anchoring, the endowment effect, and asymmetric dominance [26–28]. Substantively, understanding the process underlying inaction inertia could help consumers make less biased choices.

## 5 Experiment

Eight hundred and thirty-one students (52% female, *M*_age_ = 20.16) from a public Midwestern university participated for course credit. All materials and data are publicly archived on the Open Science Framework: https://bit.ly/3J5QW6T. This research was conducted with University of Cincinnati IRB approval (Study ID: 2014–0511). The need for written consent was waived by the ethics committee, and no minors participated in the research.

### 5.1 Basic inaction inertia effect: Likelihood of getting the special pass

**Fig 1:**
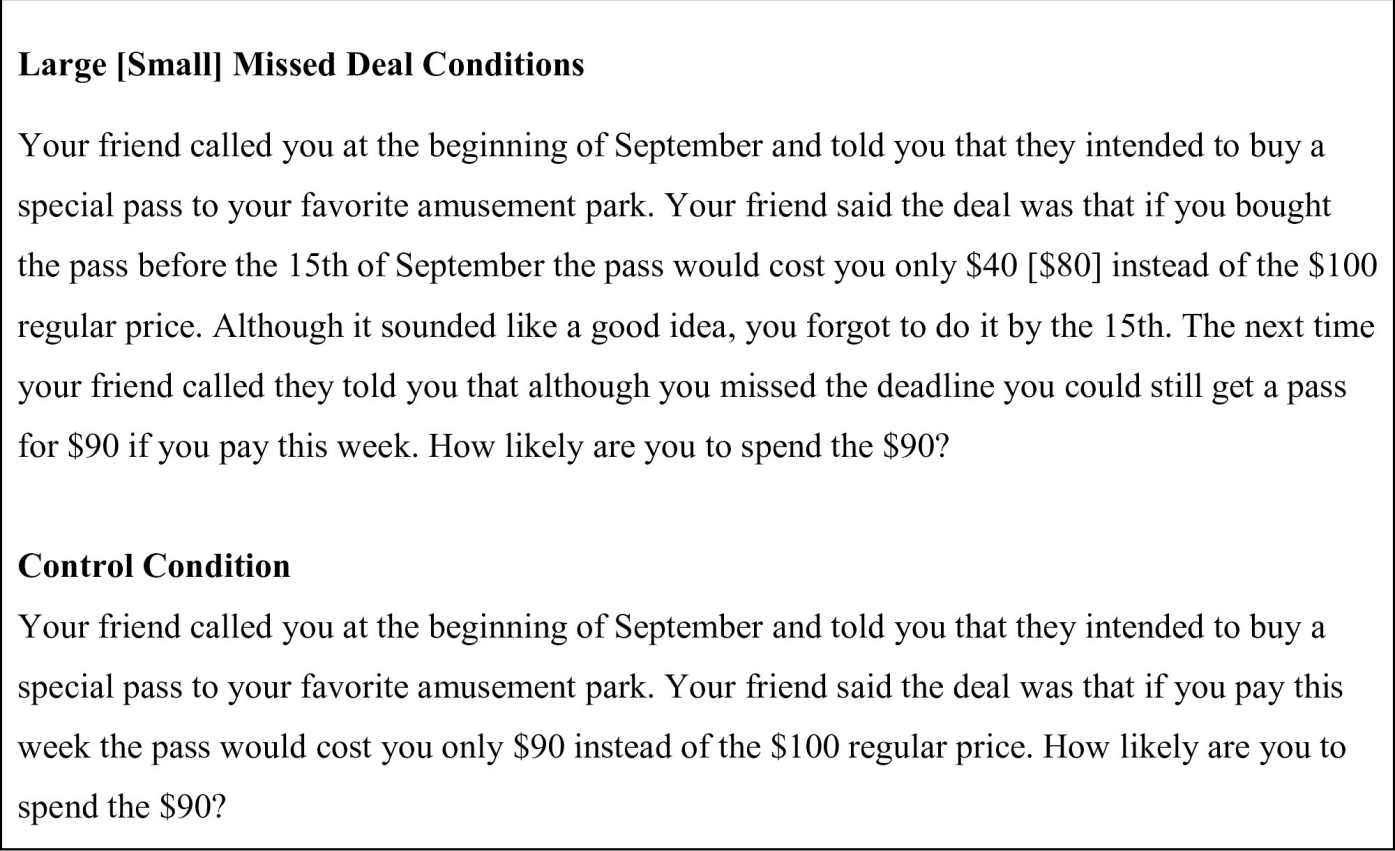
Inaction inertia scenarios.

Participants were randomly assigned to one of three conditions (larger missed deal vs. smaller missed deal vs. control) in a between-subjects design. To test our predictions we presented participants with a classic inaction inertia scenario adapted from Tykocinski et al. ([13]; see Fig 1).

This scenario was designed to fit the local season while following the design of a well-replicated inaction inertia vignette [9]. We expected to replicate the finding that participants who have missed a larger deal are less likely to take advantage of a current opportunity than those who have missed a smaller deal or those in the control condition who have no knowledge of any prior deal. In the original studies, there is no difference in likelihood of getting the special pass between the smaller deal and control conditions.

### 5.2 Thought focus and thought order measurement

Prior to the main query theory task, participants completed two practice tasks based on Johnson et al. 2007 [24] in which they were trained to list and code their thoughts about a given topic using the survey instrument. Participants were then presented with one of three inaction inertia scenarios (larger missed deal vs. smaller missed deal vs. control). To control for possible effects of the process-tracing task, participants were either instructed to list their thoughts and feelings about getting the special pass before or after indicating their likelihood of purchase. In this thought-listing ("type-out loud") protocol, participants wrote, one at a time, whatever thoughts went through their minds as they considered the decision.

Participants then indicated their likelihood of purchasing the special pass for 10% off on a scale anchored at 1 (not at all likely) and 11 (very likely). Following this, participants were shown each of the thoughts they had previously listed and were instructed to code each as a reason to get the special pass (+1), not get the special pass (-1), or neither (0). In query theory coding these are referred to as "aspects," each of which is either value-increasing (+) or value decreasing (-). We subsequently averaged the string of +1s and -1s to calculate thought focus such that -1 would represent all negative thoughts about the present deal and +1 would represent all positive thoughts. This is in contrast to typical query theory studies (e.g., Dinner et al. [29] and Johnson et al. [24]) that used the proportion of value-increasing or decreasing thoughts over total number of thoughts generated as a metric, producing a range of scores from 0 to 1. Our thought focus average maps onto this measure as follows: -1 = 0; 0 = .5; 1 = 1.

We calculated the thought order following Johnson et al. (2007) as the standardized median rank difference (SMRD) of thought types:

2(*MR*_*i*_—*MR*_*d*_)/*n* where

*MR*_*i*_ = median rank of positive thoughts about the current deal in a participant’s sequence

*MR*_*d*_ = median rank of negative thoughts in a participant’s sequence

*n* = the total number of thoughts a participant listed

Values ranged from +1 (all positive thoughts about the current deal were listed before any negative thoughts) to -1 (all negative thoughts about the current deal were listed before any positive thoughts).

### 5.3 Reference point measurement

After data collection, two independent coders blind to condition coded every thought that contained a numerical value for reference point. The coded reference point values ranged from $40 (the larger missed deal) to $100 (the usual full price of a special pass) and included $80 (the smaller missed deal). We first used an excel formula to flag any participant who mentioned any numerical value in the thought listing task. Coders then focused on these participants’ written thoughts. Thoughts that did not include numerical values were not coded. Numerical values that were ambiguous, such that coders could not determine the reference point, were also not coded. If a participant mentioned multiple numerical reference points, the values were averaged to create a reference point index for each participant who listed numerical thoughts (subsequent analysis was therefore only conducted on participants who articulated one or more thoughts that could be coded as a numerical reference point). In sum, one hundred and thirty-six participants reported codable, numeric reference point values.

### 5.4 Gain / Loss frame measurement

After data collection, the same two independent coders coded all thoughts for gain/loss frame (+1 gain / -1 loss). To someone learning about the opportunity to save $10 on their favorite amusement park pass for the first time, comparing the $100 original price with the $90 sale price should produce a gain frame, because $100 (original price)–$90 (current price) = $10 savings. Similarly, someone who has only missed a small deal of $80 should still construe the current offer as a (somewhat smaller) gain, because saving $10 rather than $20 is not dramatically different. Thus, in prior research, participants who have missed a small deal are equally as likely as those in the control condition to take advantage of the current offer. In the smaller missed deal and control conditions, a participant might write about $10 as “$10 more [than the reference point value of $80],” which would be coded as a loss (-1), or as “$10 less [than the reference point value of $100],” which, would be coded as a gain (+1). However, to someone who has missed a much larger deal, comparing the $40 previous price to the current sale price of $90 should produce a loss frame, because $40 (previous price)–$90 (current price) = $50 loss. In this case, the missed sale price of $40 –not the full price of $100 –should be the reference point, and the same $10 discount that is construed as a gain by those in the control condition should be construed as a loss. In the larger missed deal condition, participants might write about “the extra $50” [in relation to the reference point value of $40], which would be coded as a loss.

Thoughts with no clear gain/loss frame were not coded. All thoughts coded as a gain or a loss were averaged to create a Gain/Loss frame index for each participant. Subsequent analysis was therefore only conducted on participants who articulated one or more thoughts that could be coded as having a gain/loss frame. In sum, six hundred and nine participants reported codable gain/loss frame thoughts. Coder agreement was above 80% for both reference point and gain/loss frame. Disagreements were resolved through coder discussion.

## 6 Results

### 6.1 Direct effects and contrasts

#### 6.1.1 Basic inaction inertia effect: Likelihood of getting the special pass

Analysis of variance showed a significant effect of missed deal (significantly better vs. small vs. control) on likelihood of getting the special pass (*F*(2, 828) = 34.89, *p* < .001, *ηp*^2^ = .08). Follow-up contrasts showed that participants who were told they had missed a significantly better deal were less likely to take advantage of the present deal (*M* = 4.95, *SD* = 2.91) relative to those in the control condition (*M* = 6.67, *SD* = 2.87 *t*(552) = -7.00, *p* < .001, *d* = .59), or those told they had previously missed a small deal (*M* = 6.79, *SD* = 2.92; *t*(551) = -7.43, *p* < .001, *d* = .63). The small deal and control conditions did not differ (*t*(553) = -.51, *p* = .611, *d* = .04), replicating the classic inaction inertia effect. Results did not vary as a function of thought listing before versus after indicating likelihood of purchase (*F*(1, 825) = 2.57, *p* = .109, *ηp*^2^ = .003), or the interaction of thought listing by missed deal (*F*(2, 825) = .32, *p* = .724, *ηp*^2^ = .001).

#### 6.1.2 Reference point (H1)

Analysis of the reference point index–the average of all numerical reference points listed–was conducted for participants who listed relevant thoughts. Analysis of variance showed a significant effect of condition on reference point focus (*F*(2, 133) = 36.09, *p* < .001, *ηp*^2^ = .35). Follow-up contrasts revealed that participants in the larger missed deal condition focused on a much lower reference point (*M* = 65.86, *SD* = 27.29), compared to those in the control condition, whose reference point was close to the non-sale price (*M* = 97.22, *SD* = 11.76; *t*(62) = -7.11, *p* < .001, *d* = 1.47), or participants in the smaller missed deal condition (*M* = 88.08, *SD* = 9.02; *t*(54) = -5.24, *p* < .001, *d* = 1.09), supporting H1. Although not predicted, the average reference point for participants in the small missed deal condition was also significantly lower than those in the control condition (*t*(88) = 4.15, *p* < .001, *d* = .87).

#### 6.1.3 Gain / loss frame (H2)

Each thought coded as a gain or loss was averaged to create a Gain/Loss frame index for each participant. Analysis of variance showed a significant effect of missed deal on gain/loss frame (*F*(2, 606) = 38.02, *p* < .001, *ηp*^2^ = .11). Follow-up contrasts of the gain/loss index revealed that participants who were told they had previously missed a significantly better deal construed the current deal as a loss (*M* = -.43, *SD* = .78) whereas those in the control condition construed the current deal as a gain (*M* = .28, *SD* = .84; *t*(404) = -8.82, *p* < .001, *d* = .87), partially supporting H2. Not predicted, however, participants who were told they had previously missed a small deal also construed the current deal as a loss (*M* = -.14, *SD* = .86) but to a significantly lesser degree than those who had missed a much better deal (*t*(396) = -3.54, *p* < .001, *d* = .35). Also not predicted, participants who were told they had previously missed a small deal also construed the current deal as more of a loss than those in the control condition (*t*(410) = 5.02, *p* < .001, *d* = .49). Despite this, participants in the small missed deal condition were nevertheless equally as likely as those in the control condition to take advantage of the current offer.

#### 6.1.4 Thought focus and thought order (H3a and H3b)

*6*.*1*.*4*.*1 Thought focus*. Recall that the thoughts listed by each participant were coded such that -1 would represent all negative thoughts about the present deal and +1 would represent all positive thoughts. Analysis of variance showed a significant effect of missed deal on thought focus (*F*(2, 828) = 14.86, *p* < .001, *ηp*^2^ = .03). Follow-up contrasts revealed that participants who were told they had previously missed a significantly better deal produced more critical thoughts about the current deal (*M* = .001, *SD* = .63) relative to those in the control condition (*M* = .28, *SD* = .62; *t*(828) = 5.21, *p* < .001, *d* = .44), or those who only missed a small deal (*M* = .22, *SD* = .64; *t*(828) = -3.99, *p* < .001, *d* = .34), supporting H3a. As expected, the small deal and control conditions did not differ (*t*(828) = 1.22, *p* = .224, *d* = .10).

*6*.*1*.*4*.*2 Thought order*. Recall that thought order values ranged from -1 (all positive thoughts about the current deal were listed before any negative thoughts) to +1 (all negative thoughts about the current deal were listed before any positive thoughts). Analysis of variance showed a significant effect of missed deal on thought order (*F*(2, 828) = 7.99, *p <* .001, *ηp*2 = .02). Follow-up contrasts revealed that participants who were told they had previously missed a significantly better deal were more likely to generate negative thoughts about the current deal before positive ones (*M* = -.03, *SD* = .88) relative to those in the control condition (*M* = .27, *SD* = .86; *t*(552) = 3.96, *p* < .001, *d* = .34). Participants in the larger deal condition were also more likely to generate negative thoughts about the current deal before positive ones relative to the smaller deal condition (*M* = .16, *SD* = .87; *t*(551) = 2.44, *p* = .015, *d* = .21). However, there was no significant difference between those in the smaller deal and control conditions (*t*(553) = 1.51, *p* = .13). Therefore, H3b was supported.

### 6.2 Process results

#### 6.2.1 Mediating role of reference point focus (H4)

**Fig 2:**
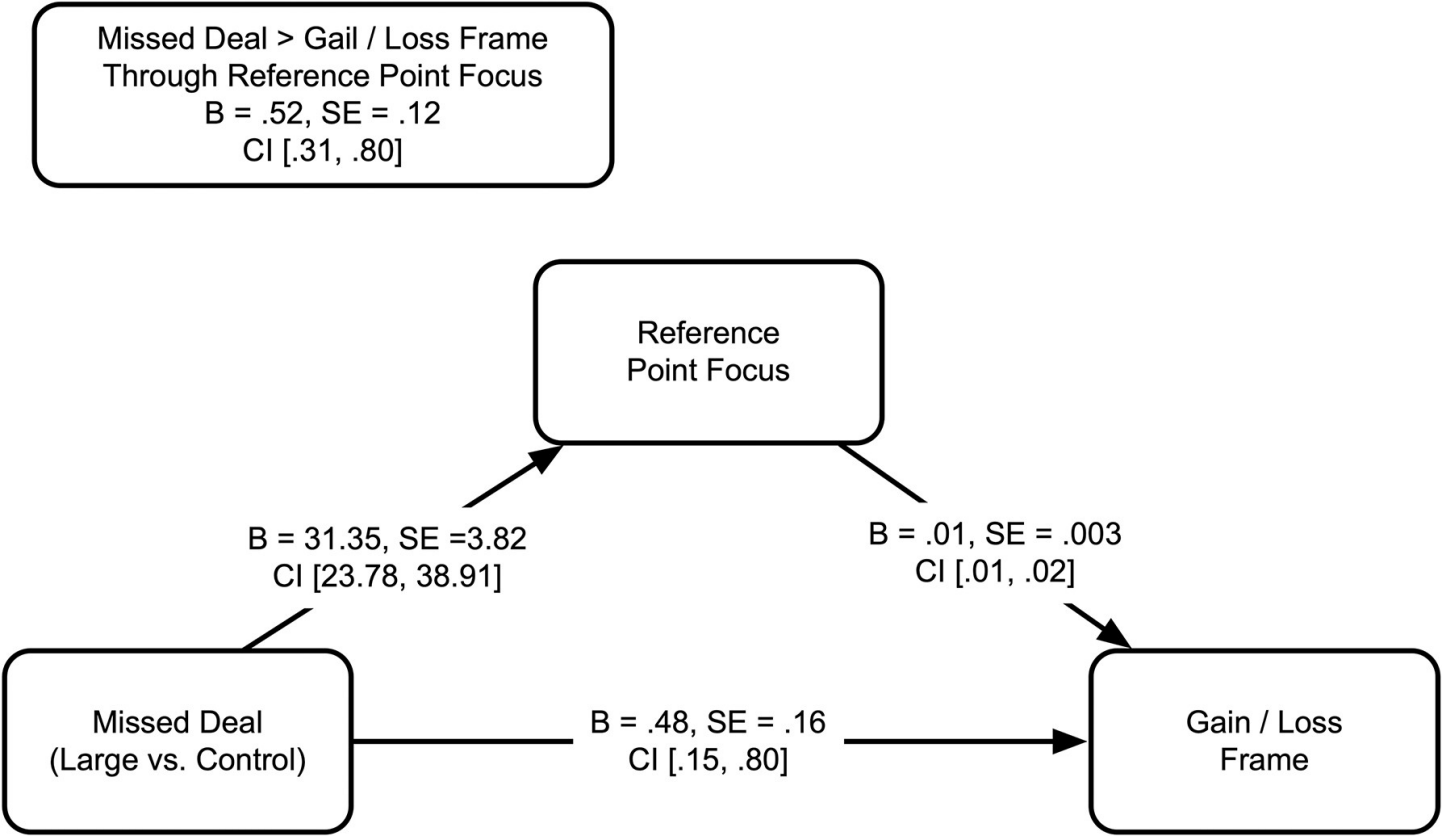
Indirect effect of missed deal on gain / loss frame through reference point focus.

We conducted mediation analysis using Hayes Process Model 4 for categorical independent variable with 5,000 bootstrapped resamples. Participants’ reference point focus mediated the effect of missed deal on perceived gain/loss (larger deal vs. control: 95% CI = [.31, .80]; smaller deal vs. control: 95% CI = [-.25, -.06]; Fig 2), supporting H4.

#### 6.2.2 Mediating role of thought focus and thought order (H5)

**Fig 3:**
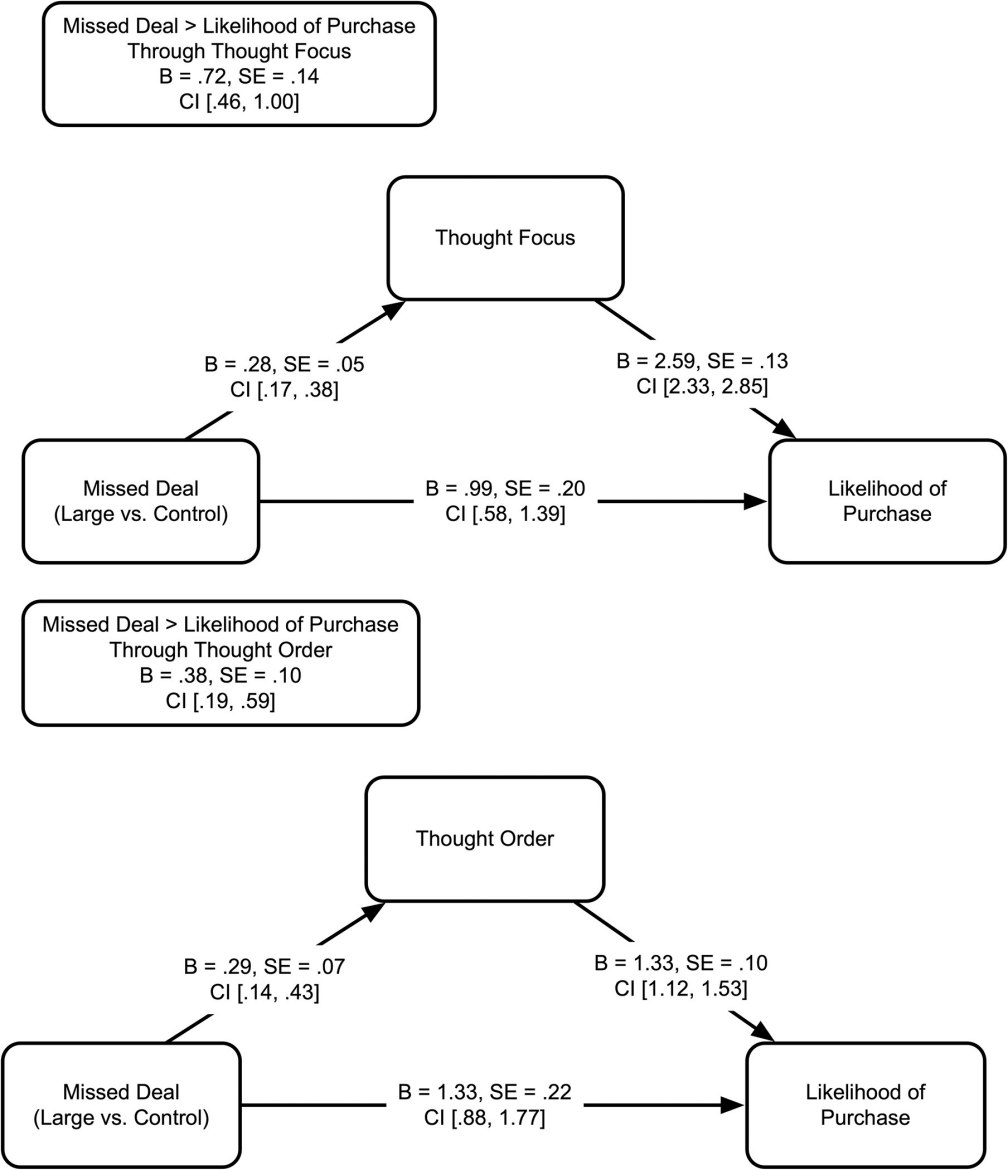
**a. Indirect effect of missed deal on likelihood of purchase through thought focus**. b. Indirect effect of missed deal on likelihood of purchase through thought order.

Mediation analysis using Hayes Process Model 4 for multicategorical independent variable with 5,000 bootstrapped resamples showed that thought focus mediated the effect of missed deal on likelihood of taking advantage of a current deal (significantly better deal vs. control: 95% CI = [.46, 1.00]; smaller deal vs. control: 95% CI = [-.44, .10]). Thought order also mediated the effect of missed deal on likelihood of taking advantage of a current deal (significantly better deal vs. control: 95% CI = [.19, .59]; smaller deal vs. control: 95% CI = [-.34, .04]), supporting H5 (Fig 3A and 3B).

### 6.3 Additional analysis comparing predictors

#### 6.3.1 Correlations among predictors

We find positive correlations among the four predictor variables (Thought Focus, Thought Order, Reference Point Index, and Gain/Loss Index). The correlations range from .10 to .66 and are expected as all four predictors are based on the same thought listings- see Table 1 below. Note that correlations including the reference point index are only calculated on the 136 participants whose thought listings included at least one numerical reference point. All other correlations are based on the entire set of respondents.

**Table 1:** Correlations of predictor variables.

	Thought Focus	Thought Order	Ref Point Index	Gain/Loss Index
Thought Focus	1	0.6632948	0.2130121	0.4390183
Thought Order	0.6632948	1	0.1085401	0.3748410
Ref Point Index	0.2130121	0.1085401	1	0.5753191
Gain/Loss Index	0.4390183	0.3748410	0.5753191	1

#### 6.3.2 Regression of choice likelihood on predictors

A regression of likelihood of getting the special pass on all four predictor variables (Thought Focus, Thought Order, Reference Point Index, and Gain/Loss Index) revealed significant effects of thought focus and gain/loss index. Participants who focused on more positive thoughts about the current deal were more likely to state that they would choose the current deal (*b* = 2.56, *t*(828) = 17.43, *p* < .001). Participants whose thoughts implied they viewed the current deal as a gain were also more likely to state they would choose the current deal (*b* = 0.35, *t*(828) = 2.79, *p* = .005). The effects of thought order and reference point index were not statistically significant (*p*s >.3).

## 7 General discussion

These results offer convergent evidence that reference dependence drives inaction inertia. As query theory and prospect theory predict, missing a large discount leads people to generate biased thoughts, which leads to biased choices. Furthermore, the significantly lower missed price creates a significantly lower reference point, so consumers view a smaller savings opportunity as a loss instead of a gain. Whereas previous accounts proposed that thinking about a superior, missed deal felt like a loss, our analysis shows that *it is the current*, *smaller deal that is construed as a loss*. Further, past work shows that missing a significantly better deal makes people devalue subsequent, smaller deals. We demonstrate a query theory process account for how this devaluation occurs–inaction inertia stems from the way consumers disproportionately generate thoughts favoring the reference option and disfavoring the alternative [24].

The current research integrates inaction inertia theory with the broader framework of reference dependence. In doing so, we bridge the gap between streams of research developed separately in the consumer behavior and judgment and decision-making literatures. Integrative theories provide generality [30] by synthesizing disparate findings about seemingly unrelated phenomena and linking them via psychological principles [31]. The field of consumer psychology needs a greater emphasis on large scale theories that synthesize and integrate a wide range of seemingly unrelated phenomena [30], moving the field towards a unified science of decision making.

This work also supplements the typical query theory analysis (coding the order and frequencies of different positive and negative thoughts) by coding and analyzing the content of the thoughts to test their consistency with prospect theory. As Johnson and Weber have pointed out [32, 33], query theory and prospect theory are not competing explanations of the phenomena they both predict (including reference dependence). Rather, these theories explain phenomena at different levels: prospect theory works at a computational level (similar to Expected Utility theory in economics), whereas query theory works at a psychological (memory) process level. In the current paper, we successfully analyze prospect theory data at a psychological process level.

Substantively, understanding that inaction inertia shares a common reference dependence mechanism with biases such as anchoring, endowment, and asymmetric dominance can help firms free consumers from inertia. Such efforts should focus on changing the focal reference point, for example by introducing new alternatives [34]. Finally, our analysis revealed that consumers who have previously missed a small deal are likely to construe the current deal as a loss even if they take advantage of the current deal. This has important implications for customer satisfaction. Ultimately, marketers should aim to offer deals that motivate customers to overcome inertia and provide the satisfaction of gaining a good value.
